# Characteristics of Newly Diagnosed Type 2 Diabetes in Chinese Older Adults: A National Prospective Cohort Study

**DOI:** 10.1155/2019/5631620

**Published:** 2019-11-15

**Authors:** Fang Lv, Xiaoling Cai, Dayi Hu, Changyu Pan, Danyi Zhang, Jie Xu, Linong Ji

**Affiliations:** ^1^Department of Endocrinology and Metabolism, Peking University People's Hospital, Beijing, China; ^2^Department of Cardiology, Peking University People's Hospital, Beijing, China; ^3^Department of Endocrinology and Metabolism, Chinese People's Liberation Army General Hospital, Beijing, China; ^4^VitalStrategic Research Institute, Shanghai, China

## Abstract

**Aim:**

To investigate the metabolic profiles of newly diagnosed type 2 diabetes (NEW2D) in Chinese older adults (≥65 years) and assess the proportion of patients who achieved the targeted goals of blood glucose, blood pressure, and blood lipid.

**Methods:**

NEW2D study was an observational, longitudinal, prospective cohort study involving patients who were diagnosed with type 2 diabetes (T2D) within the past 6 months and had a follow-up of 12 months. Participants were divided into younger NEW2D group (aged 20-65 years old) and older NEW2D group (aged ≥65 years old) according to age of diabetes onset. The baseline metabolic profiles were compared and the proportion of patients achieving adequate control of blood glucose, blood pressure, and blood lipids in reference to target goals were assessed during treatment.

**Results:**

The older NEW2D (*n* = 1362) had a lower BMI, HbA1c%, diastolic blood pressure, triglyceride, low-density lipoprotein cholesterol (LDL-C), and total cholesterol, higher systolic blood pressure, and high-density lipoprotein cholesterol levels at baseline. 47.8%, 66.7%, and 39.4% reached the target of HbA1c < 7.0%, BP < 140/90 mmHg, and LDL − C < 2.6 mmol/L, respectively. After 12 months, the proportion achieving above three targets increased to 70.2%, 76.1%, and 47.5%, respectively. The proportions of patients achieving three combined therapeutic targets doubled from 13.5% to 26.7%.

**Conclusion:**

The older NEW2D patients have special metabolic profiles compared with younger patients. The control of cardiovascular disease risk factors was suboptimal in older adults with type 2 diabetes.

## 1. Introduction

Diabetes, particularly type 2 diabetes (T2D), is becoming more prevalent in the general population, especially in older people [1]. In 2010, approximately 106 million people aged 60 years and older were living with T2D worldwide, and it is projected to increase to approximately 200 million by the year 2030 [2]. The China Non-communicable Disease Surveillance 2010 survey reported that the prevalence of diabetes in China was 22.5% and 23.5% in people aged 60-69 years and ≥70 years, respectively [3]. The estimated prevalence of newly diagnosed diabetes in older people was 14.2% to 15.5%, making the development of therapeutic strategies targeted to this broad population of patients particularly challenging [3].

Diabetes mellitus, hypertension, and dyslipidemia, which often coexist in older people, have been shown to increase the risk of cardiovascular disease (CVD) and mortality [4, 5]. However, only 29% patients aged ≥70 years and 32.8% patients aged 60-69 years are taking diabetes medications among all patients with diabetes [3]. Moreover, only 38.9% to 39.8% of patients in China treated for diabetes had adequate glycemic control [3]. Additionally, only a minority of adults with diabetes fully achieve recommended goals for glycemic control, blood pressure control, and management of dyslipidemia [6–10]. Evaluating the current control of glycemia, blood pressure, and lipid in older people with newly diagnosed type 2 diabetes (NEW2D) in China will promote a better management of risk factors of CVD in the future.

The older and younger people with newly diagnosed diabetes have unique metabolic characteristics. A cross-sectional survey of China National HbA1c Surveillance System (CNHSS) reported diabetes participants aged >40 years old had higher systolic blood pressure (SBP), higher low-density lipoprotein cholesterol (LDL-C), lower glycated hemoglobin (HbA1c), and similar BMI and triglyceride (TG) levels compared with early-onset diabetes participants (aged ≤40 years) [11]. However, the China National Diabetes and Metabolic Disorders Study reported that late-onset diabetes NEW2D had higher SBP, cholesterol (CHO), LDL-C, and 2-hour post-prandial blood glucose compared with early-onset diabetes (aged 22-43 years) [12]. However, previous studies mainly focused on the metabolic profile in early-onset T2D, though age was not defined consistently in different studies. To our knowledge, metabolic characteristics has not previously been specifically investigated in Chinese older people aged 65 years with newly diagnosis of diabetes.

Therefore, the aim of our study was to evaluate the metabolic characteristics and control of glycemia, blood pressure, and lipid in the older NEW2D in a study of the China Cardiometabolic Registry for newly diagnosed type 2 diabetic patients (CCMR-NEW2D).

## 2. Methods

### 2.1. Study Design and Population

This study was a prospective, observational cohort study with 12-month follow-up [13, 14]. From June 2012 to February 2014, patients from 81 hospitals (community hospitals (Tier 1), secondary/city level hospitals (Tier 2), and teaching or comprehensive central hospitals (Tier 3)) across six geographic regions of China (North, South, East, Southwest, Northeast, and Northwest) were recruited. Participants were enrolled at the department of endocrinology and internal medicine clinics. The inclusion criteria were as follows: (1) patients with 20 years' age or older; (2) patients with confirmed diagnosis of type 2 diabetes according to the World Health Organization criteria, within 6 months before screening; (3) patients who signed the consent form. The exclusion criteria were as follows: (1) patients who were pregnant or breastfeeding or planned to be pregnant within one year, (2) patients who were participating in another clinical trial, (3) patients who were not willing to or not able to return to the same hospital every 3 months for the follow-up visits after enrollment, and (4) patients without clear information regarding the medication used. CCMR-NEW2D study was registered at http://www.clinicaltrials.gov (NCT01525693) on February 3rd, 2012.

Ethical approval was first obtained from the Ethic committees of Peking University People's Hospital and then was approved by all the participating hospitals. All patients provided written informed consent to participate in the study prior to being screened. The research methods of the study adhered to the Declaration on Helsinki and all research was reported in accordance with strengthening the reporting of observational studies in epidemiology (STROBE) Statement.

### 2.2. Study Procedures and Data Collection

The primary objectives of NEW2D study was to assess the evolution of treatment patterns for newly diagnosed T2D patients and the clinical outcomes during 12-month follow-up in real-world condition. The patients all received routine lifestyle suggestions as diet and exercise by the investigators and also medications prescribed by the investigators. These patients were required to return to the same physician for the follow-up visits at 3, 6, 9, and 12 months after the first visit. If the patient was lost at follow-up, a structured telephone interview would be performed by the investigator to realize the patient's condition.

Physical examinations and lab tests were performed at the baseline and follow-up visits, which were described previously [13, 14]. The main clinical measurements including body mass index (BMI), waist circumference (WC), fasting plasma glucose (FPG), glycosylated hemoglobin (HbA1c), systolic and diastolic blood pressure (SBP and DBP), and total serum cholesterol (CHO, TG, LDL-C, and HDL-C). According to the Chinese Diabetes Guideline, the BMI cutoff values are categorized: BMI < 18.5 kg/m^2^ means thin, 18.5 ≤ BMI < 24 kg/m^2^ means normal, 24 ≤ BMI < 28 kg/m^2^ means overweight, and BMI ≥ 28 kg/m^2^ means obese. Target goals of blood glucose, blood pressure, and blood lipids (3Bs) were as follows: HbA1c < 7%, BP < 140/90 mmHg, and LDL − C < 2.6 mmol/L. Another more strict treatment targets of HbA1c and BP were also set for this analysis: HbA1c < 6.5% and BP < 140/80 mmHg. Another less strict treatment target of HbA1c < 7.5% was also set for this analysis. The overall proportion of patients reaching the target at baseline and follow-up were reported. For data collection and quality control, all the data were recorded in the approved case report form and entered into a web-based electronic data capture system designed by VitalStrategic Research Institute (VSRI) (Shanghai, China).

### 2.3. Statistical Analysis

Newly diagnosed diabetes participants were divided into two groups according to age: the younger NEW2D group (aged <65 years old) and the older NEW2D group (aged ≥65 years old). Descriptive statistics were used to characterize the data in the study, including calculations of means and standard deviations. The frequency and percentages (based on the nonmissing sample size) of observed levels were reported for all categorical measures. Comparisons were statistically analyzed using one-way ANOVA and chi-squared tests. *p* value <0.05 for the two-tailed test was considered statistically significant. Statistical analyses were conducted using statistical analysis system (SAS) version 9.3 (SAS Institute, Cary, North Carolina, United States of America).

## 3. Results

Totally, 5770 patients from 79 hospitals, across six geographic regions of China, were included in this study. The average age of the patients was 55.7 ± 12.6 years. Among all the newly diagnosed diabetes participants, 1362 (23.6%) participants were classified as older NEW2D.

### 3.1. Characteristics of Older NEW2D Participants

38.6%, 25.8%, and 35.5% of the older NEW2D patients were from tier 1, tier 2, and tier 3 hospitals, respectively, of which the pattern distribution was different from that in younger diabetes group (*p* < 0.0001). Older NEW2D participants had higher proportion of females and less current smokers and drinking. The older participants were more likely to take regular exercises than their younger counterparts. The older participants had lower BMI, HbA1c%, FPG, CHO, TG, LDL-C, and DBP and higher SBP and HDL-C levels compared with that of younger diabetes participants. In older NEW2D, 54.8% patients had hypertension and 41.0% had dyslipidemia at baseline. 29.0%, 15.2%, and 25.8% had comorbid hypertension, dyslipidemia, or both. Baseline characteristics of younger and older newly diagnosed type 2 diabetes participants in China were shown in Table 1.

### 3.2. Glycemic, Blood Pressure, and Blood Lipid Control

**Figure 1:**
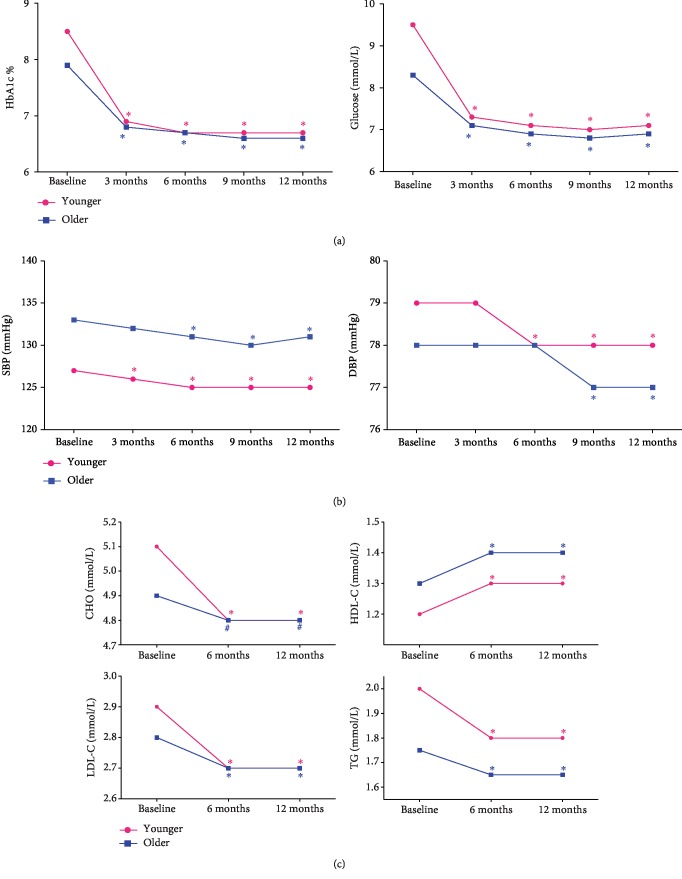
Change of blood glucose, glycosylated hemoglobin (HbA1c), blood pressure (BP), and blood lipid during 12 months follow-up. (a) Change of glycosylated hemoglobin (HbA1c) and blood glucose during follow-up. (b) Change of systolic and diastolic blood pressure during follow-up. (c) Change of blood lipid during follow-up. ^#^*p* < 0.05, ^∗^*p* < 0.01 compared to baseline.

**Figure 2:**
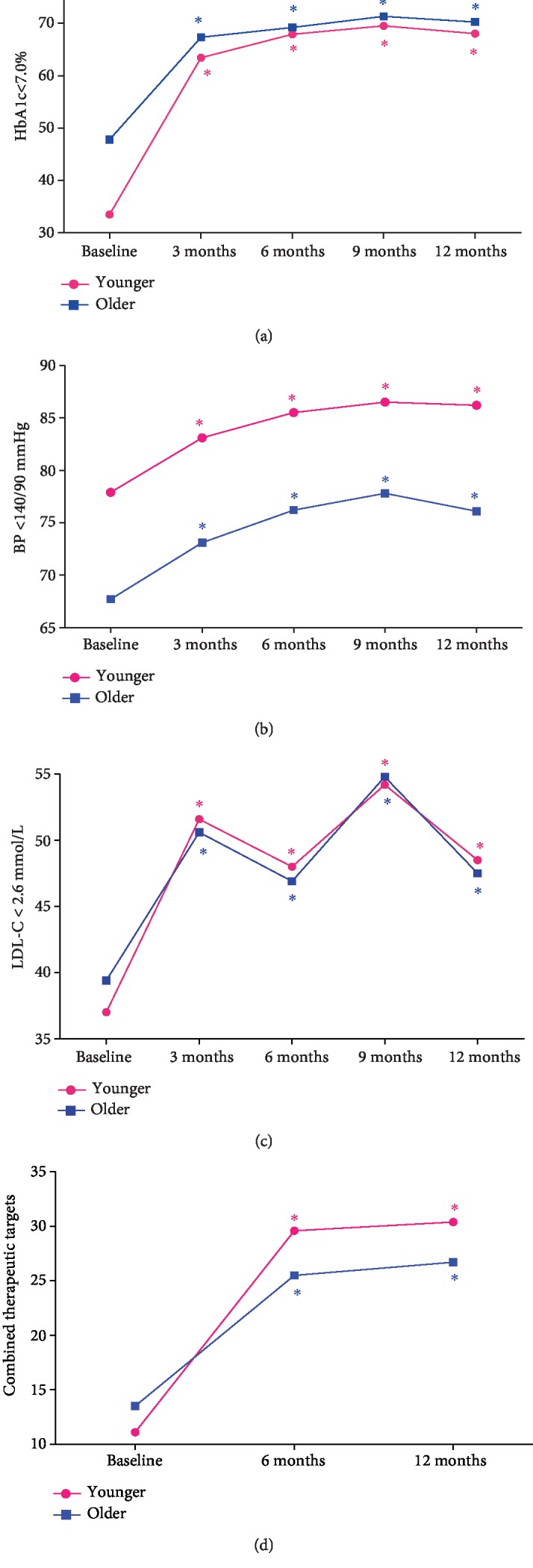
Proportions of patients achieving therapeutic targets of HbA1c < 7.0%, BP < 140/90 mmHg, and LDL − C < 2.6 mmol/L. (a) Proportions of patients achieving therapeutic targets of HbA1c < 7.0%. (b) Proportions of patients achieving therapeutic targets of BP < 140/90 mmHg. (c) Proportions of patients achieving therapeutic targets of LDL − C < 2.6 mmol/L. (d) Proportions of patients achieving three combined therapeutic targets. ^∗^*p* < 0.01 compared to baseline.

The mean HbA1c of the older NEW2D was 7.9 ± 2.4% at baseline and decreased to 6.6 ± 1.1% at 12 months. There was a significant linear downtrend of HbA1c over time (Figure 1(a)). Overall, 47.8% of the older NEW2D reached target glycemia of HbA1C < 7.0% at the baseline; 31.2% of patients reached the stricter target goal of HbA1c < 6.5%; 57.78% of patients reached the stricter target goal of HbA1c < 7.5%. By the end of 12 months follow-up, 70.2% people reached the recommended target goal of HbA1c < 7.0% (Figure 2(a)).

The mean SBP in the older NEW2D was 133 ± 15 mmHg at the baseline and slightly decreased to 131 ± 12 mmHg at 12 months (*p* < 0.0001). Similarly, the mean DBP was 78 ± 9 mmHg at the baseline and decreased to 77 ± 7 mmHg at 12 months (*p* < 0.001) (Figure 1(b)). Overall, 67.7% of the older NEW2D reached the target blood pressure (*BP* < 140/90 mmHg) at baseline and 44.9% of them reached the stricter target goal of *BP* < 140/80 mmHg (Figure 2(b)). At 12 months, the proportions of patients with adequate blood pressure control reached 76.1% using <140/90 mmHg criteria and 47% reached using <140/80 mmHg criteria.

The mean LDL-C was 2.8 ± 0.9 mmol/L at the baseline and reduced to 2.7 ± 0.9 mmol/L at 12 months (*p* < 0.0001). Overall, the proportion of the older NEW2D who achieved LDL − C < 2.6 mmol/L increased from 39.4% at baseline to 47.5% after 12 months (*p* < 0.0001) (Figure 2(c)). The mean levels of TC and TG decreased significantly after 12 months (*p* < 0.0001), and the mean level of HDL-C increased significantly after 12 months (*p* < 0.0001) (Figure 1(c)). The proportions of patients, achieving the combined therapeutic targets of HbA1c < 7.0%, BP < 140/90 mmHg, and LDL − C < 2.6 mmol/L, doubled from 13.5% to 26.7% after 12 months of treatment (*p* < 0.0001) (Figure 2(d)). And the proportion increased from 6.1% to 10.9% if the stricter control target of HbA1c < 6.5%, BP < 140/80 mmHg and LDL − C < 2.6 mmol/L (*p* < 0.0001) was used.

### 3.3. Initial Treatment Patterns

At baseline, 42.7% of the older NEW2D were on diet and exercise alone and 1.0% of them had herbal medicine only. 41.9% of the patients took OHD only (26.9% with one OHA, 13.5% with two OHAs, and 1.5% with more than two OHAs, respectively). 5.9% participating patients were treated with OHA in combination with insulin; 8.5% were treated with insulin alone. Only 16.7% of the older patients received statin therapy.

## 4. Discussion

This large, multicentre, cross-sectional, hospital-based study in China revealed that 23.6% of patients were elderly among newly diagnosed patients with diabetes. The older NEW2D in China was characterized by lower BMI, HbA1c%, FPG, CHO, TG, LDL-C, and DBP and higher SBP and HDL-C levels compared with younger patients aged less than 65 years old. The proportion of patients achieving adequate control of blood glucose, blood pressure, and blood lipids in reference to target goals improved during the 12-month follow-up, but still far from satisfactory.

It is well established that hyperglycemia, hypertension, and dyslipidemia are risk factors for microvascular diseases in T2D. Patients with T2D who had risk factor variables (such as elevated HbA1c, elevated LDL-C, albuminuria, smoking, and elevated blood pressure) within the target ranges appeared to have little or no excess risk of death, myocardial infarction, or stroke, as compared with the general population [15]. However, only 26.7% of the older NEW2D achieved the combined therapeutic targets after 12-month follow-up. This finding represents a marked improvement from previous surveys conducted in 2010, in which only 5.6% of participants achieved all triple therapeutic goals for HbA1c, BP, and CHO [9]. Studies of different countries also observed that achieving adequate control of risk factors for cardiovascular disease in NEW2D patients was challenging and were still far from satisfactory, ranging from 1.4% to 24% according to different targets of blood glucose, blood pressure, and blood lipid set by different studies [6–10].

Evidence suggests that the benefit of intensive glucose-lowering therapy is not uniform across all patients with T2D. There were few data specifically addressing optimal target HbA1c goals in older patients [16]. The widely accepted recommendation that all patients pursue HbA1c < 7.0% is based largely on the results of the UK Prospective Diabetes Study, which actively excluded people aged>65 years [17]. The results of the Action to Control Cardiovascular Risk in Diabetes (ACCORD) trial suggest that intensive therapy in persons at high risk for CVD may increase the risk for both total and CVD mortality [18]. A 5-year retrospective cohort study in the UK reported that stable glycemic level in the middle range is associated with lower risk, and more stringent targets are associated with increased mortality in people with diabetes aged 70 years and older [19]. Therefore, goals for glycemic control in older T2D should be tailored to the individual, balancing the anticipated reduction in microvascular complications over time with the possible impacts on life expectancy and risk of complications [20].

The older NEW2D patients had better glycemic control at the baseline compared to patients < 65 years of age no matter if we use the glycemic target of HbA1c < 7.0% or a more or less strict target, which was consistent with previous studies [21, 22]. Presumably, older people had better adherence to the lifestyle program compared with the younger age groups. Moreover, the older NEW2D mostly resemble mild age-related diabetes (MARD), who only had modest metabolic derangements [23, 24]. A real-world population suggested the rate of deterioration in those diagnosed at over 70 years of age was very low, with 66% having a rate of deterioration of less than 1.1 mmol/mol HbA1c per year [25]. In addition, older adults (>65 years) with diabetes are at risk of developing a similar spectrum of microvascular complications as their younger counterparts with diabetes, albeit probably at lower absolute risk if they develop their diabetes later in life, which will limit duration. Therefore, treatment in individuals diagnosed older may not need to be as aggressive as those diagnosed younger.

LDL-C reduction was associated with the greatest CVD risk reduction, followed by blood pressure and glycemic control [5]. However, in NEW2D STUDY, only little progress has made with regard to hyperlipidemia management during 12 months follow-up, with just 47.5% of older NEW2D having their LDL-C levels adequately controlled, and only 18.4% having statin treatment at 12 months. Recent guidelines suggested that moderate-intensity statin therapy should be started in patients 40 to 75 years of age with diabetes mellitus and LDL − C ≥ 1.8 mmol/L if there is no contraindication without calculating 10-year atherosclerotic cardiovascular disease (ASCVD) risk [26]. This pattern represented a gap from the clinical guidelines and indicated that improvement in the quality of lipid management were needed in patients with T2D in the real world.

There are some limitations of this study. First, given the limitations of observational research and our short follow-up time, the clinical implication of the observed characteristics in the older NEW2D participants was not known. Prospective cohort studies with longer follow-up are necessary to observe the natural history of newly diagnosed diabetes in Chinese older patients. Secondly, the HbA1c target for older patients was still controversial. However, as this study was an observational cohort study, with the aim to evaluate clinical outcomes and glycemic control in the real world in China, we did not stratify the target of glucose control for different ages in this study. Randomized, prospective trials that have examined glycemic control and complications focusing on the older patient (>65 years) are needed to obtain the optimal glycemic target.

## 5. Conclusions

The older NEW2D patients have special metabolic profiles and higher proportion of comorbid hypertension and hyperlipidemia. The proportion achieving adequate control of risk factors for cardiovascular disease in older NEW2D patients were improved during treatment. However, control of glycemia, BP, and LDL were far from optimal despite the widespread use of guidelines for the management of diabetes and CVD. Awareness and application of published recommendations need to be reinforced.

## Figures and Tables

**Table 1:** Characteristics of younger and older groups of newly diagnosed diabetes participants at baseline.

	Onset age of diabetes			
	Younger NEW2D group (aged<65 years old)	Older NEW2D group (aged≥65 years old)	*p* value	Total
Total, *N* (%)	4408 (76.4)	1362 (23.6)		5770 (100.0)
Age (mean ± SD)	50.64 ± 9.63	71.90 ± 5.05	**<0.001**	55.66 ± 12.59
Female, *N* (%)	1923 (43.63)	717 (52.64)	**<0.001**	2640 (45.8)
Hospital classification				
1st tier	838 (19.0)	526 (38.6)	**<0.001**	1364 (23.6)
2nd tier	1225 (27.8)	352 (25.8)	0.148	1577 (27.3)
3rd tier	2345 (53.2)	484 (35.5)	**<0.001**	2829 (49.0)
Positive family history of diabetes, *N* (%)	1440 (32.67)	188 (13.80)	**<0.001**	1628 (28.2)
Current smoking, *N* (%)	1146 (26.00)	125 (9.18)	**<0.001**	1271 (22.0%)
Current drinking, *N* (%)	542 (12.30)	78 (5.73)	**<0.001**	620 (10.75)
Regular exercises, *N* (%)	1485 (33.69)	531 (38.99)	**<0.001**	2016 (34.9)
BMI (mean ± SD)	25.17 ± 3.47	24.57 ± 3.19	**<0.001**	25.03 ± 3.41
Percentage with BMI < 24 kg/m^2^	1650 (37.4)	599 (44.0)	**<0.001**	2249 (39.0)
Percentage with 24.0 ≤ BMI < 28 kg/m^2^	1959 (44.4)	585 (43.0)	0.363	2544 (44.1)
Percentage with BMI ≥ 28 kg/m^2^	799 (18.1)	178 (13.1)	**<0.001**	977 (16.9)
T2D only	1679 (38.1)	409 (30.0)	**<0.001**	2088
T2D+HTN	617 (14.0)	395 (29.0)	**<0.001**	1012
T2D+DYLP	1323 (30.0)	207 (15.2)	**<0.001**	1530
T2D+HTN+DYLP	789 (17.9)	351 (25.8)	**<0.001**	1140
Family CVD history	902 (20.46)	165 (12.11)	**<0.001**	1067 (18.49)
FPG (mmol/L)	9.5 ± 3.9	8.3 ± 3.5	**<0.001**	9.2 ± 3.9
HbA1c (%)	8.5 ± 2.5	7.9 ± 2.4	**<0.001**	8.4 ± 2.5
Percentage with HbA1c < 7.5%	1904 (43.74)	772 (57.78)	**<0.001**	2676 (47.04)
Percentage with HbA1c < 7.0%	1458 (33.5)	638 (47.8)	**<0.001**	2096 (36.8)
Percentage with HbA1c < 6.5%	955 (21.9)	417 (31.2)	**<0.001**	1372 (24.1)
Mean SBPs (mmHg)	127 ± 14	133 ± 15	**<0.001**	129 ± 14
Mean DBPs (mmHg)	79 ± 9	78 ± 9	**<0.001**	79 ± 9
Percentage with BP < 140/80 mmHg	1953 (44.3)	612 (44.9)	0.683	2565 (44.5)
Percentage with BP < 140/90 mmHg	3436 (77.9)	922 (67.7)	**<0.001**	4358 (75.5)
CHO (mmol/L)	5.1 ± 1.3	4.9 ± 1.2	**<0.001**	5.0 ± 1.3
HDL-C (mmol/L)	1.2 ± 0.4	1.3 ± 0.3	**<0.001**	1.2 ± 0.4
TG (mmol/L)	2.4 ± 2.1	1.9 ± 1.4	**<0.001**	2.4 ± 11.6
LDL-C (mmol/L)	2.9 ± 1.0	2.8 ± 0.9	**0.001**	2.9 ± 1.0
Percentage with LDL − C < 2.6 mmol/L	1608 (37.0)	531 (39.4)	0.116	2139 (37.6)
HbA1c < 7.5%, blood pressure < 140/90 mmHg, and LDL − c < 2.6 mmol/L	614 (14.26)	215 (16.19)	0.082	829 (14.71)
HbA1c < 7.0%, blood pressure < 140/90 mmHg, and LDL − c < 2.6 mmol/L	476 (11.1)	179 (13.5)	**0.016**	655 (11.6)
HbA1c < 6.5%, blood pressure < 140/80 mmHg, and LDL − c < 2.6 mmol/L	220 (5.1)	81 (6.1)	0.161	301 (5.3)

1st tier: community hospitals; 2nd tier: secondary/city level hospitals; 3rd tier: teaching or comprehensive central hospitals (tier 3); BMI: body mass index; SBP: systolic blood pressure; DBP: diastolic blood pressure; CHO: cholesterol; LDL-C: low-density lipoprotein cholesterol; HDL-C: high-density lipoprotein cholesterol; FPG: fasting plasma glucose; T2D: diabetes; HTN: hypertension; DYLP: dyslipidemia.

## Data Availability

The data that support the findings of this study are available from VitalStrategic Research Institute (Shanghai, China) but restrictions apply to the availability of these data, which were used under license for the current study, and so are not publicly available. Data are however available from the authors upon reasonable request and with permission of VitalStrategic Research Institute (Shanghai, China).
